# *iMutSig*: a web application to identify the most similar mutational signature using shiny

**DOI:** 10.12688/f1000research.24435.2

**Published:** 2020-11-19

**Authors:** Zhi Yang, Priyatama Pandey, Paul Marjoram, Kimberly D. Siegmund

**Affiliations:** 1 Department of Preventive Medicine, Keck School of Medicine of the University of Southern California, 2001 N.Soto Street, Los Angeles, CA, 91003, USA

**Keywords:** Mutational Signatures, pmsignature, COSMIC, Web interface, Shiny, R

## Abstract

There are two frameworks for characterizing mutational signatures which are commonly used to describe the nucleotide patterns that arise from mutational processes. Estimated mutational signatures from fitting these two methods in human cancer can be found online, in the Catalogue Of Somatic Mutations In Cancer (COSMIC) website or a GitHub repository. The two frameworks make differing assumptions regarding independence of base pairs and for that reason may produce different results. Consequently, there is a need to compare and contrast the results of the two methods, but no such tool currently exists. In this paper, we provide a simple and intuitive interface that allows comparisons of pairs of mutational signatures to be easily performed. Cosine similarity measures the extent of signature similarity. To compare mutational signatures of different formats, one signature type (COSMIC or *pmsignature*) is converted to the format of the other before the signatures are compared. *iMutSig* provides a simple and user-friendly web application allowing researchers to download published mutational signatures of either type and to compare signatures from COSMIC to those from *pmsignature*, and vice versa. Furthermore, *iMutSig* allows users to input a self-defined mutational signature and examine its similarity to published signatures from both data sources. *iMutSig* is accessible online and source code is available for download from GitHub.

## Introduction

Each human is subject to a variety of mutational processes throughout their lifetime. These processes result in a catalog of somatic mutations in the tissue creating a unique mutational profile ^1^. A mutational signature captures the pattern of the mutations and contexts in which those mutations occur (i.e., the neighboring bases). Examples of important mutational processes with distinct mutational signatures include aging and ultraviolet (UV) radiation. Additionally, many research groups are performing analysis to discover *de novo* mutational signatures in cancer ^1– 4^.

Currently, there are two frameworks used to characterize and visualize mutational signatures ^5, 6^. The first, proposed by Alexandrov *et al.*, uses a vector of 96 probabilities to capture the composition of the six nucleotide substitutions (C *>*A, C *>*T, C *>*G, T *>*A, T *>*C, T *>*G) and the neighboring base immediately on each of the 5′ and 3′ side of the mutated base ^1^. A list of published mutational signatures can be downloaded from the Catalogue Of Somatic Mutations In Cancer (COSMIC) website ^7^ (version 2, v2). Later, Alexandrov *et al.* published an expanded set of mutational signatures in version 3.1 (v3.1) ^8^. The 72 COSMIC v3.1 Single Base Substitution (SBS) signatures include 30 v2 signatures. Based on the signature concept, but using different model assumptions, Shiraishi *et al.* proposed a mixed-membership model, *pmsignature*, which substantially reduced the number of parameters needed to characterize a signature ^9^. They achieved this by assuming independence across bases, thereby reducing the number of parameters from 6*4*4-1 = 95 to (6-1)+(4-1)+(4-1) = 11 ^9^. The reduction in the number of parameters is greater if more flanking bases are included. However, the independence assumption might prevent signatures with dependent neighboring bases from being discovered, thereby resulting a fewer signatures. Shiraishi identified 27 signatures, all of which can be downloaded from their GitHub repository ^9^. In this paper, we will refer to signatures resulting from these two methods as “COSMIC signatures” with version numbers (for those resulting from Alexandrov *et al.*’s method) and “PM signatures” (for those resulting from Shiraishi *et al.*’s method).

A large number of researchers have published scientific findings resulting from the COSMIC signature-based method ^10– 12^, which was defined as the “gold standard" in the field by Baez-Ortega *et al.* ^6^. Meanwhile, an increasing number of researchers are using the *pmsignature*-based method for samples with lower numbers of somatic variants due to it requiring fewer parameters ^9, 13, 14^. Given that both methods are widely used, investigators need the ability to compare results from their analysis with those reported in earlier databases, which may have been produced using the alternate method. For example, researchers have adopted both tools for gastric cancer and tried to compare and integrate the information from two data sources in a somewhat *ad hoc* manner ^15^. No rigorous tool exists for this task. In this paper we present *iMutSig*, an easy-to-use tool that allows users to 1) input a new mutational signature, 2) compare it using cosine similarity to all published signatures from both the COSMIC and PM signature databases, 3) identify the most similar signatures previously reported, and 4) to assemble the information characterizing those signatures using simple point-and-click navigation.

## Methods

### Implementation

**Figure 1.**
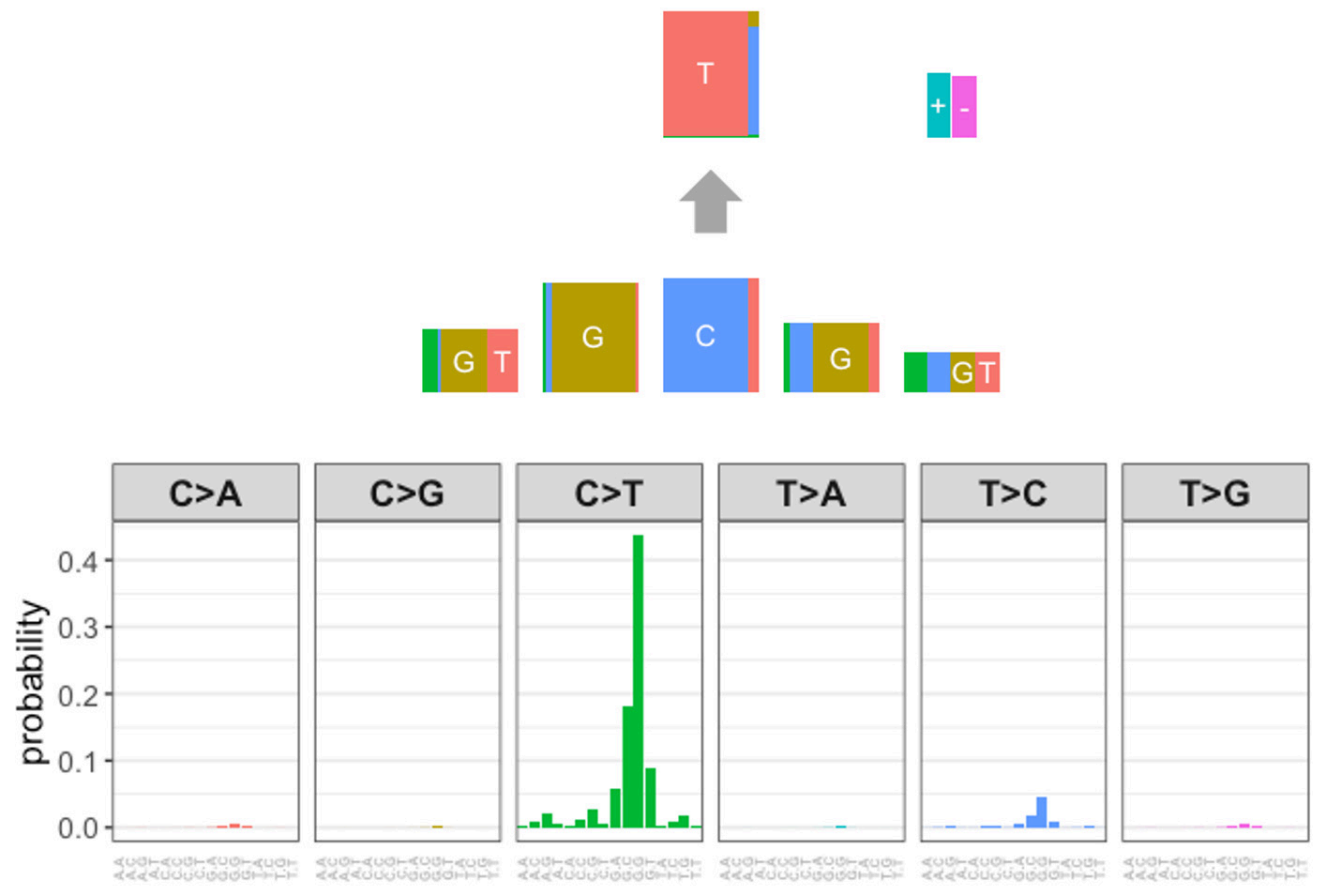
The PM signature appearing in Table 1 (top) with the ‘expanded’ signature appearing in COSMIC format (bottom).

In order to measure the similarity between mutational signatures across two databases, we need to represent PM signatures in a way that is comparable with those from COSMIC, or represent COSMIC signatures in a way comparable to PM signatures. We call the first of these methods the “expand” method, where we expand the PM signature into a probabilistic vector with the same length as the COSMIC signature, i.e., 96. The conversion in the opposite direction, from the COSMIC signature into the PM signature format is called the “collapse” method. In the collapsed format, the PM signature is represented by a vector of 14 probabilities, the probabilities for the six possible nucleotide substitutions and the probabilities for the four possible bases at each of the two flanking base positions. In the “expand” method, to calculate each of 96 resulting probabilities in the vector, we take the constituent components that make up the COSMIC signature - which refer to the nucleotide substitution and two flanking bases at the -1 and +1 position - calculate the probability of each component for the given PM signature, and then multiply those probabilities using PM signature’s assumption of independence. For example, to calculate the probability of the COSMIC signature C[C >A]T we multiply three PM signature’s probabilities: *P*(C at pos -1), *P*(C >A), and *P*(T at pos +1). This example is shown in Table 1, Equation 1, and Figure 1.

**Table 1.** An example of PM signatures.

Nucleotide substitution						
C>A	C>G	C>T	T>A	T>C	T>G
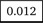	0.003	0.879	0.003	0.090	0.014
Flanking bases					
Position	A	C	G	T	
-2	0.159	0.042	0.486	0.314	
-1	0.044	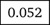	0.870	0.034	
+1	0.076	0.237	0.571	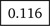	
+2	0.245	0.247	0.256	0.252	
Transcription strand					
Plus			Minus		
0.511			0.489		

$$\begin{array}{l} P(C[C\;>A]T)=P(C\;\text{at}\;\text{pos}\;-1)P([C\;>A])P(T\;\text{at}\;\text{pos}\;+1)\\ \;\;\;\;\;\;\;\;\;\;\;\;\;\;\;\;\;\;\;\;\;\;\;\;\;\;\;\;\;\;=0.052\times \;0.012\;\times \;0.116\\ \;\;\;\;\;\;\;\;\;\;\;\;\;\;\;\;\;\;\;\;\;\;\;\;\;\;\;\;\;\;=7.24\;\times \;10^{-5} \end{array}\;(1)$$

To perform the “collapse” method, we calculate the marginal probability for each characteristic, the nucleotide substitution and each flanking base, and multiply the probabilities together using the independence assumption. The marginal probability for the nucleotide substitution is computed by summing the probabilities including all 16 combinations of two flanking bases from the COSMIC signature. In a similar manner, the marginal probability of a flanking base is the sum of probabilities across all signatures containing the given flanking base. See an example of P(C>A) and P(C at pos -1) shown in Equation 2:

$$\begin{array}{l} \;P(C\;>A)=\sum_{i}^{A,C,G,T}\sum_{j}^{A,C,G,T}P(i[C>A]j)\\ P(C\;\text{at}\;\text{pos}\;-1)=\sum_{j}^{A,C,G,T}\sum_{i}^{C>A,C>G,C>T,T>A,T>C,T>G}P(C[i]j) \end{array}\;(2)$$

These are computed using the *convertAlexandrov2Shiraishi* function from the *decompTumor2Sig* package ^15^.

After we have represented both forms of signature using probabilistic vectors of the same length *n*, ****P**** and ****C**** say, we can directly compare the two signature types. In order to measure the similarity between them we use cosine similarity, *CS*, defined as shown in Equation 3:

$$CS(P,C)=\frac{P\cdot C}{\left\|P\right\|\cdot \left\|C\right\|}=\frac{\sum_{i=i}^{n}P_{i}\cdot C_{i}}{\sqrt{\sum_{i=1}^{n}P_{i}^{2}}\cdot \sqrt{\sum_{i=1}^{n}C_{i}^{2}}}\;(3)$$

Intuitively speaking, cosine similarity is the cosine of the angle between the two vectors. As such, cosine similarity ranges from 0 to 1 (inclusive). In our context, if two mutational signatures have a cosine similarity of 1, they must be identical, i.e., the angle between them is 0°; in contrast, if two mutational signatures have a cosine similarity of 0, they are maximally dissimilar (i.e., orthogonal). Computing the cosine similarity between the input signature and each of the candidate signatures, and then sorting the similarities from highest to lowest value, we identify the candidate signature with the highest cosine similarity as the most similar mutational signature.

### Operation

**Figure 2.**
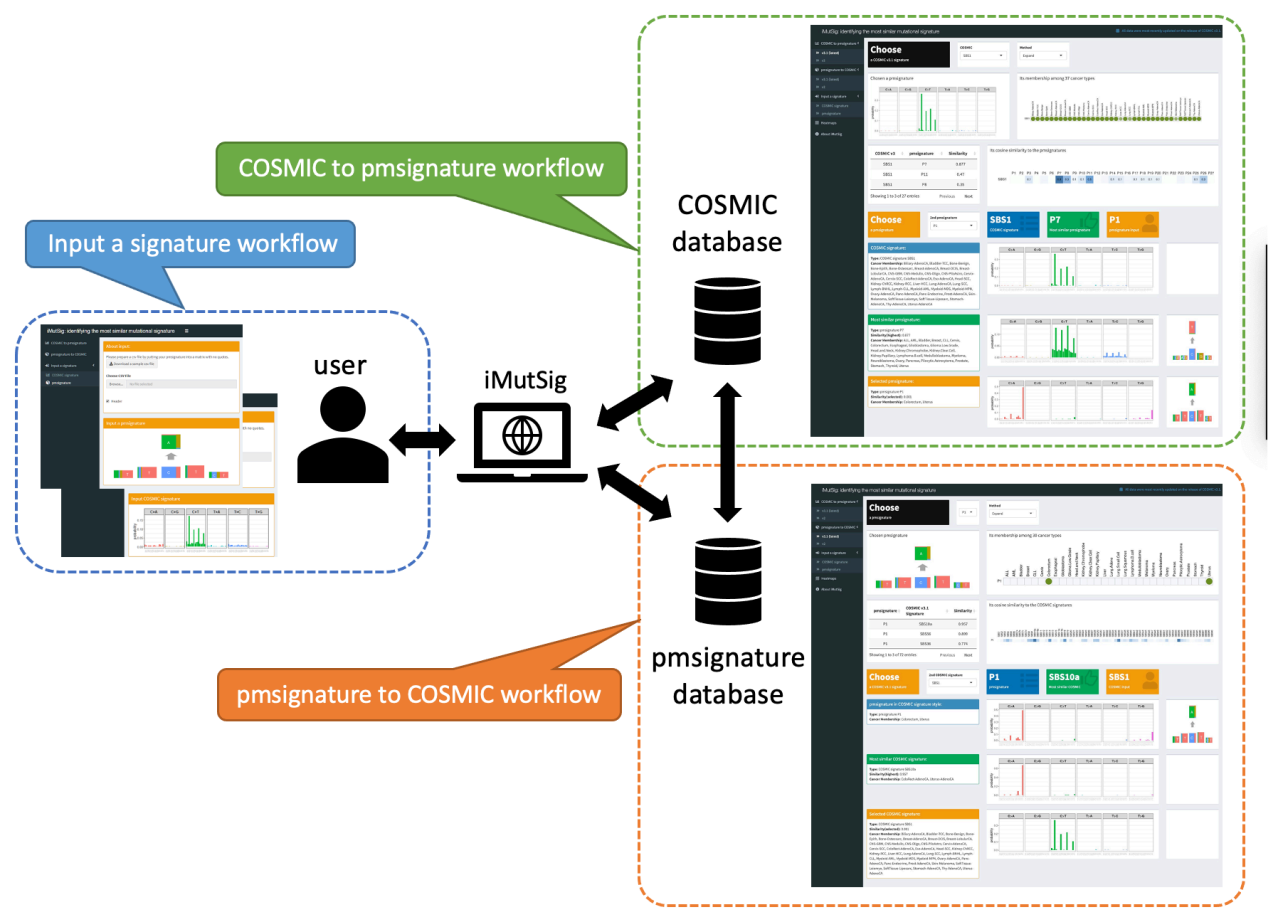
Overview of three workflows in the *iMutSig* interface. The first two tabs allow users to finding the most similar PM signature to an input COSMIC signature (highlighted in green) and vice versa (highlighted in orange). In addition, users can identify the most similar signatures from both data sources to an input signature (highlighted in blue).

*iMutSig* is built in R with its key features depending on the R package, *pmsignature* ^9^. As shown in Figure 2, the Shiny app currently supports three possible workflows for users to choose from, depending on the type of signatures they have already obtained: 1) starting with a COSMIC signature; 2) starting with a PM signature; 3) starting with a self-defined signature that could follow either the COSMIC or PM format.

The first tab in the Shiny app window, “COSMIC to pmsignature", allows users to select an input COSMIC signature via a drop-down list and returns the best-matched PM signature. The returned results are divided and organized separately in the top and the bottom portion of the page. The top half tab summarizes background information regarding the input signature by presenting: 1) visualized plots of the input signature and its membership among all cancer types, i.e., in which kind of cancers the mutational signatures has been found; 2) a table showing the cosine similarity between this signature and all PM signatures, sorted in decreasing order, along with a visualization of a similarity heatmap with color and intensity proportional to assessed similarity. The bottom half tab presents plots and descriptions of the input COSMIC signature, the most similar PM signature, and a second PM signature that the user can select. Thus, users can easily access all the vital information and results regarding these signatures rather than having to manually gather and organize information from publications. The top half of the tab will be automatically updated via a control panel in the middle section of the tab, which enables users to select a signature to start with and also highlights information about the currently selected signature, the most-similar signature from the alternate model framework, and the cosine similarity.

The second tab was designed in a similar manner to the first tab, but for the case in which we are starting with a PM signature and looking for the most similar COSMIC signature. For the first two tabs, users can choose which version of COSMIC signatures to input from the sub-menus, i.e., v2 or v3.1.

Unlike the first two tabs, the third tab enables users to enter a user-supplied signature, which can be in either PM or COSMIC format, and then identify the most similar signature from each online database. The user will be requested to enter a sub-menu based on the type of the input signature and to upload a comma-separated values (CSV) file containing a single signature. A sample CSV file is provided for download to give the user a better sense of the format of the input file. Then, the tab will be updated to display three tables, one from each data source (COSMIC v2, v3.1 and PM), listing the signatures from that data source and the cosine similarity of each signature with the user-uploaded signature. The tables are ordered from most similar to least similar signature. In addition, the user is able to view figures of the best-matched signatures (i.e., those with highest cosine similarity) from each data source, allowing users to observe any similarities and dissimilarities. Below, users will see a list of cancer types that contain the best-matched signature.

**Figure 3.**
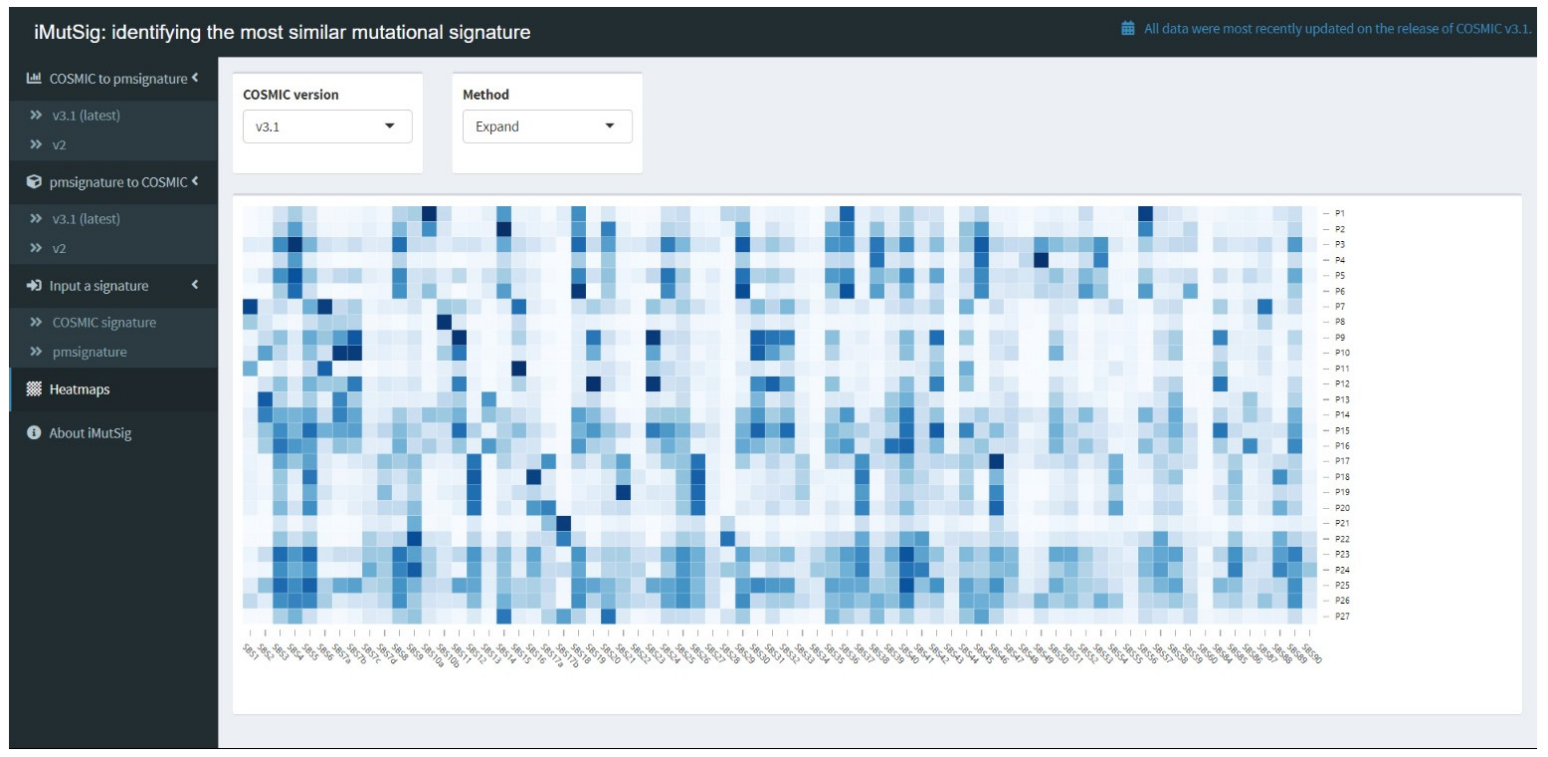
Cosine similarity heatmaps between PM signatures and COSMIC signatures.

The fourth tab shown in Figure 3 displays the interactive cosine similarity heatmaps between PM signatures and COSMIC signatures for the two conversion methods. One would choose the version of COSMIC signatures (v2 or v3.1) and one of the two conversion methods (COSMIC to PM signature, ‘collapse’, or PM signature to COSMIC, ‘expand’). The PM signature, the COSMIC signature names and the associated cosine similarity value can be visualized by placing the cursor over the heatmap. It is notable that the cosine similarity values tend to be higher using the collapse representation compared to the expand representation. We attribute this to the difference in model assumptions. When a COSMIC signature is collapsed to the PM signature format the independence assumption is imposed on both signature types. However, when a PM signature is expanded to the COSMIC signature format, the PM signature probability vector still represents the fit under feature independence whereas the COSMIC signature does not. This difference in model assumptions results in lower estimates of cosine similarity. Some discrepancies are found, based on the conversion method selected, when searching for the most similar signature from the opposite database: matching COSMIC v3.1 signatures to PM signatures 17 out of 72 disagreed (23.6%). A similar fraction disagreed when matching COSMIC v2 to PM signatures (7 out of 30, 23.3%). Interestingly, when we compare the 27 PM signatures to COSMIC, we see much better agreement with the newer v3.1 signatures compared to the earlier v2 signatures (88.9% vs 63%). The higher matching of the v3.1 database includes the matching of signatures that were not present in the earlier v2 database (e.g. SBS10b, SBS46, SBS49). The remaining discrepant results may correspond to COSMIC signatures that reflect dependence between neighboring bases. 

## Use cases

**Figure 4.**
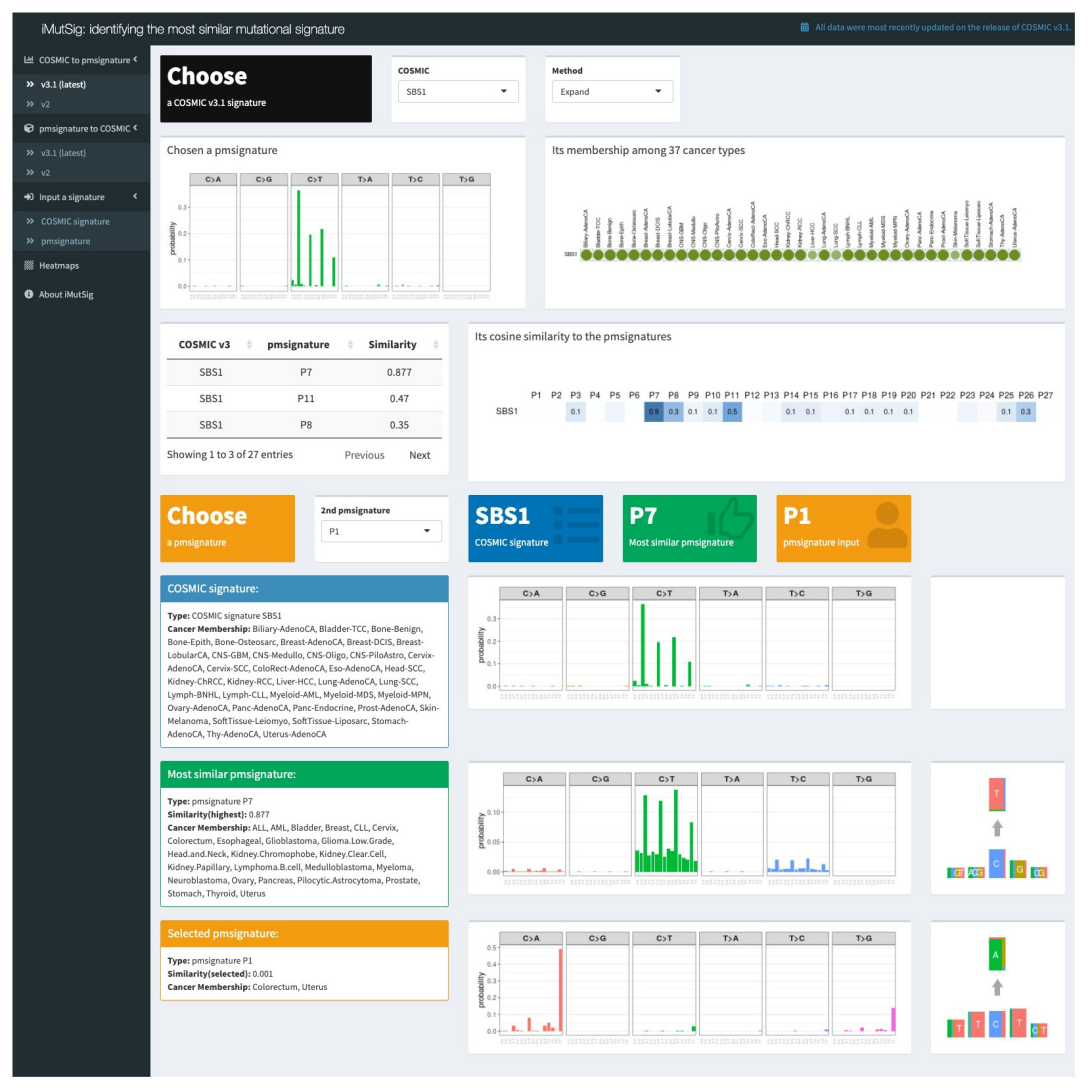
Input a COSMIC v3.1 signature, SBS1.

**Figure 5.**
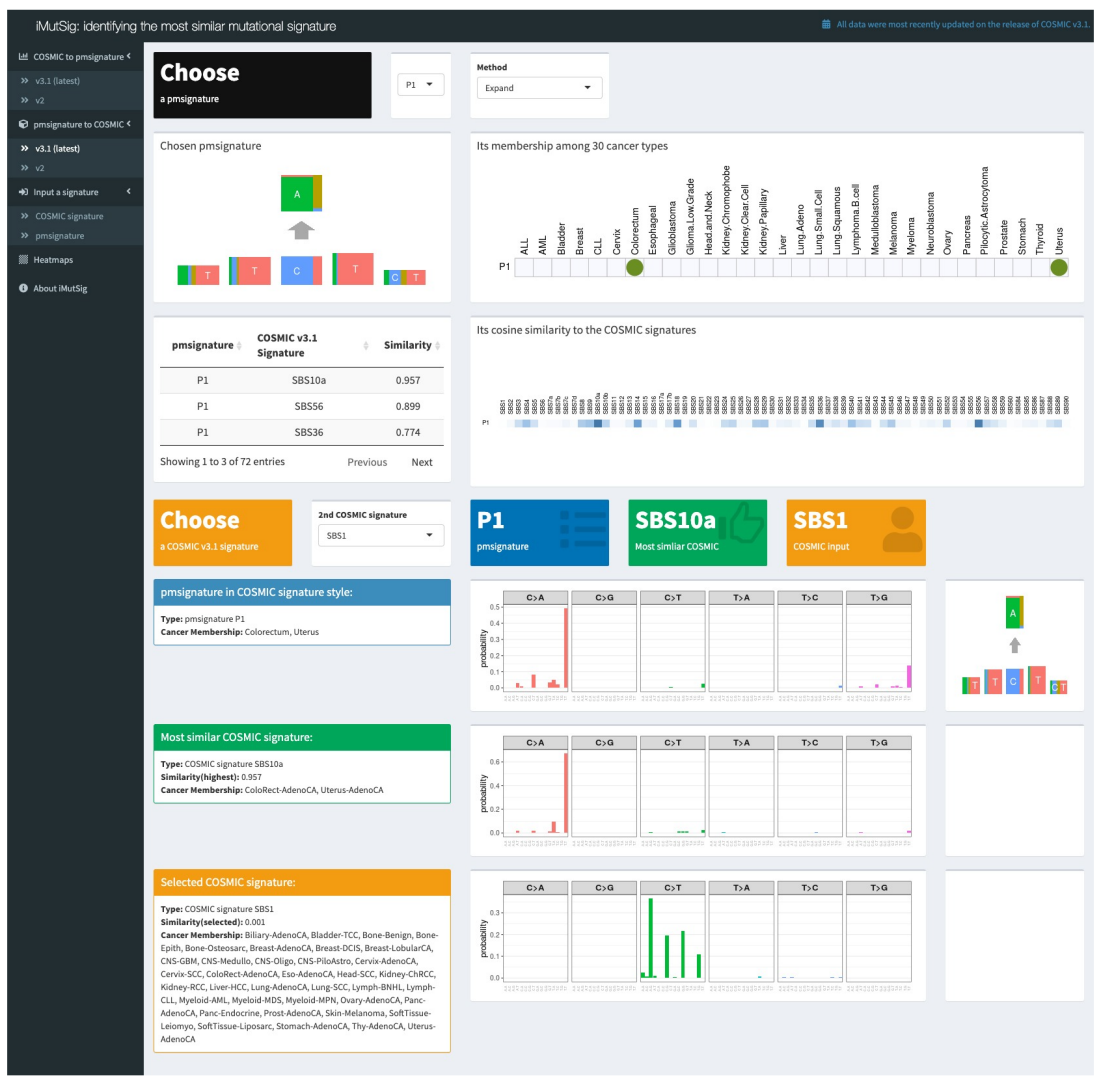
Input a PM signature, P1.

**Figure 6.**
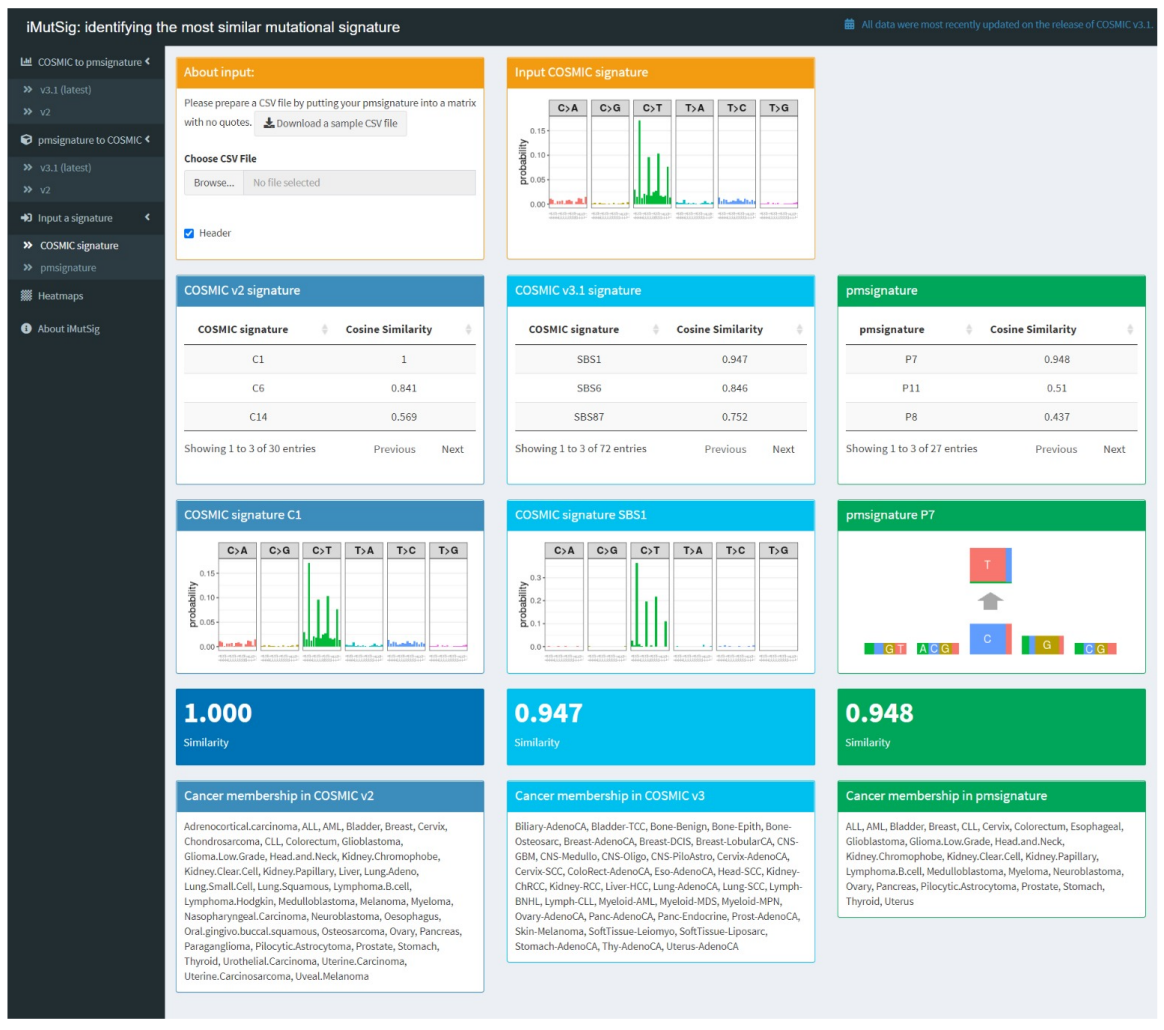
Input a user-supplied COSMIC signature.

**Figure 7.**
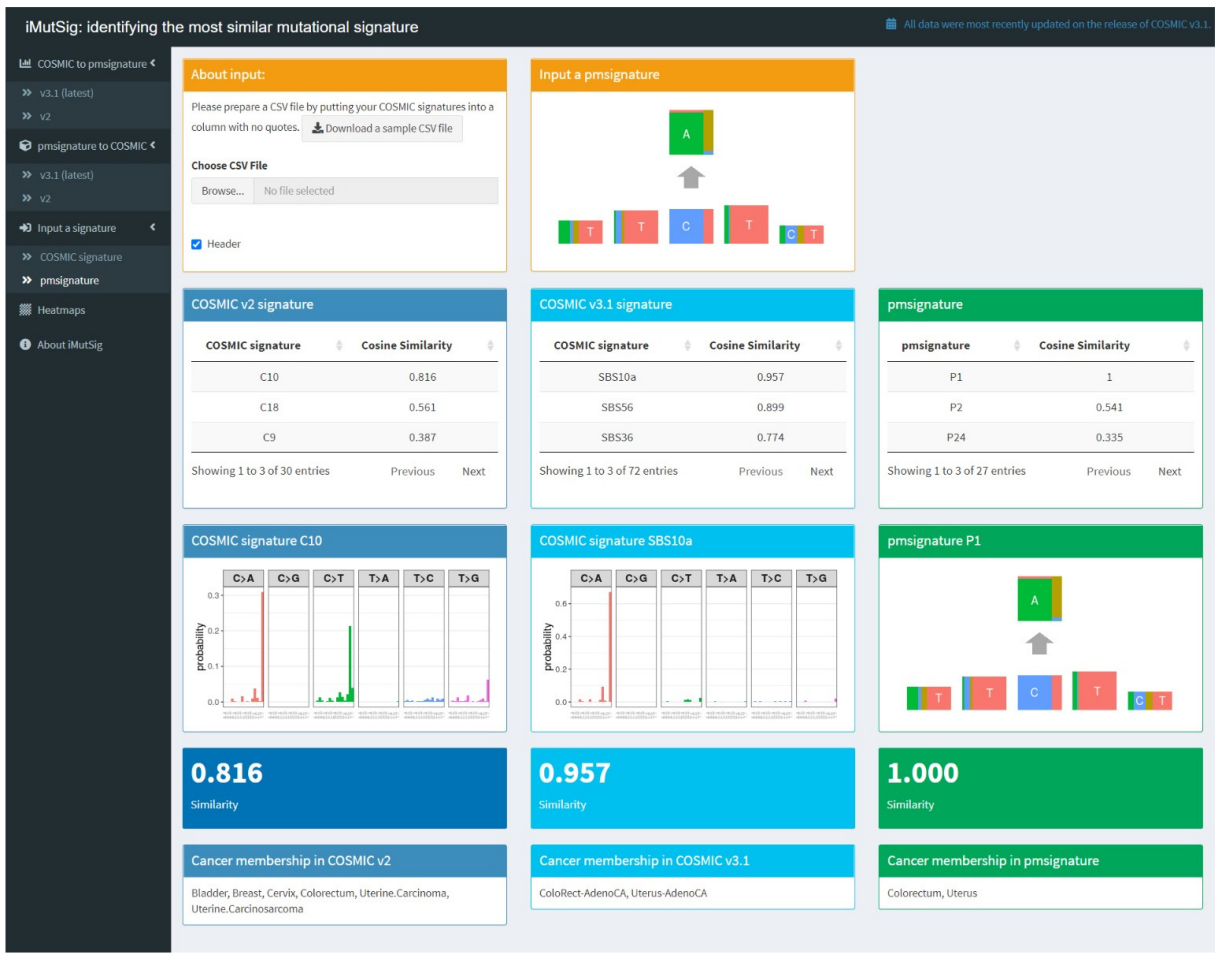
Input a user-supplied PM signature.

We use *iMutSig* to identify the most similar signature for a given PM/COSMIC signature or a user-supplied signature. Figure 4 shows the input panel after inputting COSMIC v3.1 signature SBS1 and Figure 5 shows the input panel after inputting PM signature P1. If users provide a user-supplied signature of either COSMIC-kind or PM-kind, the results can be seen in Figure 6 and Figure 7. Consider the example shown in Figure 6, where we input COSMIC v2 signature C1. *iMutSig* returned the most similar signatures COSMIC v3.1 signature SBS1, and PM signature P7 (similarity = 0.947, and 0.948, respectively) along with the names of its associated cancer types. When providing PM signature P1, *iMutSig* returned COSMIC v2 signature C10, v3.1 signature C10a and PM signature P1 (similarity = 0.816, 0.957, 1.0, respectively).

## Conclusions

*iMutSig* is a user-friendly interactive browser-based application that allows users who have a signature that they have discovered in an analysis of their own data to identify the best-matched existing mutational signature from the COSMIC and PM databases. It also allows users to directly compare signatures between the two databases. It does this in an interactive way, and also allows straightforward visualization of results. *iMutSig* enables researchers to easily identify the most similar mutational signature and to easily access characteristic information from both data sources without additional software installation and programming of their own.

## Data availability

All data underlying the results are available as part of the article and no additional source data are required.

## Software availability

Software available from: https://zhiyang.shinyapps.io/iMutSig/

Source code available from: http://www.github.com/USCbiostats/iMutSig

Archived source code at time of publication: https://doi.org/10.5281/zenodo.4132416 ^16^

License: MIT
