# Supplementary material for: iMutSig: a web application to identify the most similar mutational signature using shiny
Source: F1000Res. 2020 Nov 19;9:586. Originally published 2020 Jun 10. [Version 2] doi: 10.12688/f1000research.24435.2 (PMC7702159; doi:10.12688/f1000research.24435.2)
Supplement: Peer review history

# Peer review: Reviewer response for version 2

**DOI:** 10.5256/f1000research.30641.r75096

**Published:** 2020-11-27

**Authors:** Adrian Baez-Ortega

**Affiliations:** 1 Transmissible Cancer Group, Department of Veterinary Medicine, University of Cambridge, Cambridge, UK

In this revised manuscript, Yang *et al*. satisfactorily address all the major and minor revisions originally requested, resulting in improvements to both the paper and the software tool. Hence I believe this paper to be scientifically sound in its present form, and no further major revisions should be necessary. However, below I add a few non-essential points that could be addressed, all of which are quite straightforward and should not require an additional round of review. Moreover, I would understand if the authors disagreed with my last comment regarding cosine similarities.

1. In the iMutSig app, I noticed that the panel showing the membership of the selected COSMIC signature across tumour types does not display anything for some of the v3.1 signatures (e.g. SBS27, SBS28), while it does work for their v2 counterparts (e.g. C27, C28). I do not know if this is a mistake or if the distributions of some v3.1 signatures are really unknown.
2. Introduction, par. 2 reads: "thereby resulting a fewer signatures" instead of "thereby resulting **in** fewer signatures"; and "Shiraishi identified 27 signatures" instead of "Shiraishi ***et al*.** identified 27 signatures".
3. In Implementation, par. 1, when discussing the "expand" method whereby a COSMIC signature is constructed from three of the probabilities in the input PM signature, perhaps it would be good to mention that the PM signature may contain more features than just substitution type and immediate 5' and 3' bases (such as probabilities for -2/+2 bases and transcriptional strand), and that any information about these extra features of the PM signature is lost when "expanding" to the COSMIC format. Adding this comment might be valuable since the example in Table 1 does contain these extra features, and it would also highlight one of the strengths of PM signatures (more information in less parameters).
4. Implementation, par. 1 reads: "For example, to calculate the probability of the COSMIC signature C[C >A]T...". Should it not be "the COSMIC **mutation type **C[C>A]T"? A COSMIC signature would be a vector of 96 such mutation types/categories.
5. The comment above also applies to the next paragraph, which reads: "In a similar manner, the marginal probability of a flanking base is the sum of probabilities across all **signatures** containing the given flanking base."
6. Implementation, par. 2 reads: "To perform the “collapse” method, we calculate the marginal probability for each characteristic, the nucleotide substitution and each flanking base, and **multiply the probabilities together** using the independence assumption". Are you sure that the probabilities are multiplied together in the "collapse" method? As far as I understand, each marginal probability is obtained as a summation (as shown in Equation 2), and no multiplication is required afterwards – if I am correct, these marginal probabilities already define the PM signature.
7. Figure 2 legend reads: "The first two tabs allow users to finding...", instead of "to **find**".
8. Operation, par. 5 reads: "It is notable that the cosine similarity values tend to be higher using the collapse representation compared to the expand representation. We attribute this to the difference in model assumptions. When a COSMIC signature is collapsed to the PM signature format the independence assumption is imposed on both signature types. However, when a PM signature is expanded to the COSMIC signature format, the PM signature probability vector still represents the fit under feature independence whereas the COSMIC signature does not." While I understand this, I am not sure this is the only reason for the difference in cosine similarities. In general, one would expect high cosine similarity values to become less frequent as the number of dimensions increases; in other words, it is much less likely to find two 96-dimensional vectors with a near-zero angle between them than it is to find two 11-dimensional vectors with a similar angle. So it is possible that the mere fact of collapsing a COSMIC signature into a PM signature causes an overall increase in its cosine similarity with every other PM signature (and the opposite would be true when "expanding" signatures). However, I do not know which of these two explanations is more important for the change in similarity values observed in the signature heatmaps.

Are the conclusions about the tool and its performance adequately supported by the findings presented in the article?

Yes

Is the rationale for developing the new software tool clearly explained?

Yes

Is the description of the software tool technically sound?

Partly

Are sufficient details of the code, methods and analysis (if applicable) provided to allow replication of the software development and its use by others?

Yes

Is sufficient information provided to allow interpretation of the expected output datasets and any results generated using the tool?

Yes

Reviewer Expertise:

Computational biology, Bioinformatics.

I confirm that I have read this submission and believe that I have an appropriate level of expertise to confirm that it is of an acceptable scientific standard.

# Peer review: Reviewer response for version 2

**DOI:** 10.5256/f1000research.30641.r75097

**Published:** 2020-11-26

**Authors:** Vittorio Perduca

**Affiliations:** 1 Université de Paris, CNRS, MAP5 UMR 8145, F-75006, Paris, France

Thank you for taking into account my comments and for clarifying the differences between the original "expand" method and the new "collapse" method. I have no further comments.

Are the conclusions about the tool and its performance adequately supported by the findings presented in the article?

Partly

Is the rationale for developing the new software tool clearly explained?

Yes

Is the description of the software tool technically sound?

Partly

Are sufficient details of the code, methods and analysis (if applicable) provided to allow replication of the software development and its use by others?

Yes

Is sufficient information provided to allow interpretation of the expected output datasets and any results generated using the tool?

Yes

Reviewer Expertise:

Applied statistics, biostatistics.

I confirm that I have read this submission and believe that I have an appropriate level of expertise to confirm that it is of an acceptable scientific standard.

# Peer review: Reviewer response for version 1

**DOI:** 10.5256/f1000research.26954.r64570

**Published:** 2020-07-15

**Authors:** Vittorio Perduca

**Affiliations:** 1 Université de Paris, CNRS, MAP5 UMR 8145, F-75006, Paris, France

This paper presents an original online tool for comparing mutational signatures represented according to two alternative formats, namely COSMIC vectors with the relative frequencies of the 96 types of substitutions on one side ^1^, and lower dimensional "pmsignature" vectors on the other side ^2^. The article is well written and the method behind the tool is clearly explained. The interactive tool runs smoothly and has the potential to provide useful support to researchers running mutational signature analyses using alternative frameworks.

 My comments:

- One important point worth stressing is that the proposed solution for comparing "PM signatures" and "COSMIC signatures" is to represent the former lower dimensional probabilistic vectors in the larger space of the latter vectors. This is clearly explained in the methods, but I believe it is worth mentioning explicitly that this happens even when the input is of the COSMIC type.
- My major concern is that the described method relies on the pmsignature assumption of independence between the mutation features. Could the authors comment about the possible limitations entailed by this assumption?
- In my understanding, another possibility would have been to represent "COSMIC signatures" as 11-dimensional "PM signatures" by eliminating through summation two features out of three. For instance, one could compute the "PM signature" component representing the probability of the substitution S=[C>A] as 
  P(S = [C>A]) = sum_{l, r} P(L=l, S=[C>A], R=r), where L and R denote the -1 and +1 flanking bases, and P(L=l, S=[C>A], R=r) is one of the 96 probabilities in the input "COSMIC signature". This approach does not rely on the pmsignature assumption of independence. (This solution does not make it possible to consider "PM signatures" with more than two flanking bases, but in any case the information about such extra bases is lost when converting "PM signatures" to "COSMIC signatures" using the method described in the paper). Have the authors explored this other method? A comment on this point could possibly help clarifying the reason why the authors have decided to rely on the independence assumption.
- I suggest to add a figure with the heatmap showing the cosine similarity between the original 27 "PM signatures" and the 30 v2 "COSMIC signatures". This would help understanding the type of correspondence between the two databases.

Minor point:

- "Implementation" paragraph, line 6: pm-signature -> pmsignature.

Are the conclusions about the tool and its performance adequately supported by the findings presented in the article?

Partly

Is the rationale for developing the new software tool clearly explained?

Yes

Is the description of the software tool technically sound?

Partly

Are sufficient details of the code, methods and analysis (if applicable) provided to allow replication of the software development and its use by others?

Yes

Is sufficient information provided to allow interpretation of the expected output datasets and any results generated using the tool?

Yes

Reviewer Expertise:

Applied statistics, biostatistics.

I confirm that I have read this submission and believe that I have an appropriate level of expertise to confirm that it is of an acceptable scientific standard, however I have significant reservations, as outlined above.

## References

[1] : Signatures of mutational processes in human cancer. *Nature*.2013;500(7463) : 10.1038/nature12477 415-21 10.1038/nature12477 23945592PMC3776390

[2] : A Simple Model-Based Approach to Inferring and Visualizing Cancer Mutation Signatures. *PLoS Genet*.2015;11(12) : 10.1371/journal.pgen.1005657 e1005657 10.1371/journal.pgen.1005657 26630308PMC4667891

# Response

**Published:** 2020-11-05

**Authors:** Zhi Yang

**Affiliations:** University of Southern California, USA

This paper presents an original online tool for comparing mutational signatures represented according to two alternative formats, namely COSMIC vectors with the relative frequencies of the 96 types of substitutions on one side ^1^, and lower dimensional "pmsignature" vectors on the other side ^2^. The article is well written and the method behind the tool is clearly explained. The interactive tool runs smoothly and has the potential to provide useful support to researchers running mutational signature analyses using alternative frameworks.

 My comments:

- One important point worth stressing is that the proposed solution for comparing "PM signatures" and "COSMIC signatures" is to represent the former lower dimensional probabilistic vectors in the larger space of the latter vectors. This is clearly explained in the methods, but I believe it is worth mentioning explicitly that this happens even when the input is of the COSMIC type.

**Response**: We now summarize explicitly the tasks the software performs at the end of the introduction: “ *iMutSig*, an easy-to-use tool that allows users to 1) input a new mutational signature, 2) compare it using Cosine similarity to all published signatures from both the COSMIC and PM-signature databases, 3) identify the most similar signatures previously reported, and 4) to assemble the information characterizing those signatures using simple point-and-click navigation.”

- My major concern is that the described method relies on the pmsignature assumption of independence between the mutation features. Could the authors comment about the possible limitations entailed by this assumption?

**Response**: We are not recommending one method over another, but are providing a means for comparing signatures that are estimated under the different modeling assumptions. One can transform the format of either signature type to the format used by the other signature type (PM signature to COSMIC or COSMIC to PM signature). In the original submission we only considered expanding the PM signature to the COSMIC format. In this revision we have added the capability of collapsing the COSMIC signature to the PM signature format (see response to next comment). These are not symmetric activities and can lead to differences in the identification of most similar signature from the opposite model. The new method of ‘collapsing’ the COSMIC signature is described in the section: “Methods/Implementation”. The consequences for identifying the most similar signature of the opposite type are described in the last paragraph under “Methods/Operation”.

- In my understanding, another possibility would have been to represent "COSMIC signatures" as 11-dimensional "PM signatures" by eliminating through summation two features out of three. For instance, one could compute the "PM signature" component representing the probability of the substitution S=[C>A] as 
  P(S = [C>A]) = sum_{l, r} P(L=l, S=[C>A], R=r), where L and R denote the -1 and +1 flanking bases, and P(L=l, S=[C>A], R=r) is one of the 96 probabilities in the input "COSMIC signature". This approach does not rely on the pmsignature assumption of independence. (This solution does not make it possible to consider "PM signatures" with more than two flanking bases, but in any case the information about such extra bases is lost when converting "PM signatures" to "COSMIC signatures" using the method described in the paper). Have the authors explored this other method? A comment on this point could possibly help clarifying the reason why the authors have decided to rely on the independence assumption.

**Response**: Thank you for this suggestion. As we noted in our response to your last point, we have added this additional comparison to the app using a function implemented in the decompTumor2Sig package. Interestingly, the cosine similarity values tend to be higher using the above method. We interpret this in the paper as follows: “When a COSMIC signature is collapsed to the PM signature format the independence assumption is imposed on both signature types. However, when a PM signature is expanded to the COSMIC signature format, the PM signature probability vector still represents the fit under feature independence whereas the COSMIC signature does not. This difference in model assumptions results in lower estimates of cosine similarity.” (section Methods/Operation).

- I suggest to add a figure with the heatmap showing the cosine similarity between the original 27 "PM signatures" and the 30 v2 "COSMIC signatures". This would help understanding the type of correspondence between the two databases.

**Response**: The heatmap is now added to the Shiny app and the manuscript.

 Minor point:

- "Implementation" paragraph, line 6: pm-signature -> pmsignature.

**Response**: We have corrected this typo in this version of the manuscript.

# Peer review: Reviewer response for version 1

**DOI:** 10.5256/f1000research.26954.r64568

**Published:** 2020-06-22

**Authors:** Adrian Baez-Ortega

**Affiliations:** 1 Transmissible Cancer Group, Department of Veterinary Medicine, University of Cambridge, Cambridge, UK

Yang *et al*. present an interactive software tool, iMutSig, which allows comparison between two alternative mathematical representations of mutational signatures. Both of these representations are widely used, but are remarkably different in their visual aspect, making intuitive comparisons difficult. To my knowledge, this is the first openly available method for comparison between signatures expressed in these two alternative representations.

 The methods implemented for conversion between signature representations and for comparison between signatures are straightforward and based on a widely used similarity measure; although the main formula in the paper is incorrect, this mistake does not appear to extend to the implementation. Instead of simply reporting the most similar signature to the chosen signature, the tool provides information about the similarity of the chosen signature to all the signatures available in the alternative representation, allowing better assessment of the results. The user interface is thoughtfully and tastefully designed, making the tool both easy and pleasant to use. All the offered functionalities appear to work correctly and the platform runs smoothly. The authors provide their tool as an interactive website, as well as current and archived versions of the source code. However, there is a lack of information about how to install and run the software locally as an R package, which would increase the long-term usability of the tool.

 Below I provide comments regarding major and minor issues in the article and online tool. I also provide a few optional suggestions that may be safely ignored, but which I think would enhance the functionality of the tool.

**MAJOR COMMENTS**

**1.** Implementation, paragraph 2: The formula for the cosine similarity defined in Equation 2 is not correct. While it is true that

 CS(P,C) = (P·C) / (||P||·||C||),

 it is not true that

 CS(P,C) = sum(P_i * C_i) / (sum(P_i) * sum(C_i)).

 The correct formula for the third part of Equation 2 would be:

 CS(P,C) = sum(P_i * C_i) / (sqrt(sum(P_i^2)) * sqrt(sum(C_i^2)))

 (see here).

 Please ammend this formula, and make sure that it gives the same values as the formula you have defined in your code (function getCosDistance).

**MINOR COMMENTS**

**2.** Introduction, paragraph 1: This is somewhat inaccurate, in the sense that it is the cells in an organism's tissues that are exposed to mutational processes throughout the organism's life, and each cell or tissue develops its own mutational profile. The existence of a "unique mutational profile" thus may be better described as a property of a tissue, organ or tumour: it is not really accurate to say that each human has "his/her unique mutational profile", as this varies widely across tissues (e.g. cells in the blood, skin, liver, and colon have very different mutational spectra), and the differences among individuals also tend to be tissue-specific.

**3.** Introduction, paragraph 3: in the sentence where the motivation for PM signatures is mentioned ("due to it requiring fewer parameters"), it might be appropriate to add a brief note that the assumption of independence between substitution type and flanking bases also limits the representation of patterns where these features are dependent, although this is seen in relatively few COSMIC signatures (e.g. SBS8, SBS25, SBS35).

**4.** Implementation, paragraph 1: it would be useful to complement the example in Table 1 and Equation 1 with a figure that shows the original PM signature in Table 1, and the equivalent COSMIC signature that results from applying Equation 1 to each of the 96 substitution types. This would help the reader to understand the conversion between both graphical representations, which is shown in later figures.

**5. **Note that reference 5 has an updated version: Omichessan, Severi & Perduca (2019) ^1^.

**6.** Note that reference 6 is missing a colon between the author list and title.

**7.** The "About iMutSig" web page states that "On the Github page, you can: - install the iMutSig R pacakge and run it locally." However, on the GitHub page I found no instructions on how the package can be installed and run locally using R and Shiny. While I understand that the main purpose of the platform is to be an online tool, some users may be interested in having a local copy. For example, the availability of the tool seemed somewhat variable: I was able to access the website (https://zhiyang.shinyapps.io/imutsig/) on 17 June, but not on 18 June. Although this might be a rare issue, it highlights the advantage of providing users with an alternative way of accessing the tool in the long term. For example, some simple steps for installation and running could be added as a README.md file on the GitHub repository.

**8.** Note that, in the platform interface, some of the signature names read "COSIMIC" instead of "COSMIC".

**OPTIONAL SUGGESTIONS**

**9.** I found it strange that in the tabs "COSMIC to pmsignature" and "pmsignature to COSMIC", the drop-down menus for choosing the COSMIC and PM signatures to compare are located in the middle of the interface, below the top panels that show the chosen signature. It seems to me that it would be more intuitive to place the selection menus at the top of the page, although I might be wrong.

**10.** The authors might consider extending the user-supplied signature mode to allow the user to input a set of signatures, and then select the signature to compare using a drop-down menu (as in the other comparison modes), as it is likely that users will be interested in analysing sets of signatures, rather than single signatures. However, I understand this might not be straightforward to implement.

**11.** Another potential extension could be an additional mode in which comparison could be performed between two sets of user-supplied signatures (each of which could be in COSMIC or PM format), in order to find the best one-to-one match between the signatures.

Are the conclusions about the tool and its performance adequately supported by the findings presented in the article?

Yes

Is the rationale for developing the new software tool clearly explained?

Yes

Is the description of the software tool technically sound?

Partly

Are sufficient details of the code, methods and analysis (if applicable) provided to allow replication of the software development and its use by others?

Yes

Is sufficient information provided to allow interpretation of the expected output datasets and any results generated using the tool?

Yes

Reviewer Expertise:

Computational biology, Bioinformatics.

I confirm that I have read this submission and believe that I have an appropriate level of expertise to confirm that it is of an acceptable scientific standard, however I have significant reservations, as outlined above.

## References

[1] : Computational tools to detect signatures of mutational processes in DNA from tumours: A review and empirical comparison of performance. *PLoS One*.2019;14(9) : 10.1371/journal.pone.0221235 e0221235 10.1371/journal.pone.0221235 31513583PMC6741849

# Response

**Published:** 2020-11-05

**Authors:** Zhi Yang

**Affiliations:** University of Southern California, USA

We are very grateful to the reviewers for their comments and suggestions. We give detailed responses to each of those comments below.

**MAJOR COMMENTS**

**1.** Implementation, paragraph 2: The formula for the cosine similarity defined in Equation 2 is not correct. While it is true that

 CS(P,C) = (P·C) / (||P||·||C||),

 it is not true that

 CS(P,C) = sum(P_i * C_i) / (sum(P_i) * sum(C_i)).

 The correct formula for the third part of Equation 2 would be:

 CS(P,C) = sum(P_i * C_i) / (sqrt(sum(P_i^2)) * sqrt(sum(C_i^2)))

 (see here).

 Please amend this formula, and make sure that it gives the same values as the formula you have defined in your code (function getCosDistance).

**Response**: The formula has been corrected in this new version of the manuscript. We have also verified that the formula was correctly implemented in the getCosDistance function in the software (see below).

 getCosDistance <- function(F_1, F_2) {

   if(length(F_1)!=length(F_2)){

     geterrmessage("Two signatures have different number of bases!")

   }

   cos <- sum(F_1*F_2)/(sqrt(sum(F_1^2))*sqrt(sum(F_2^2)))

   return(cos)

 }

**MINOR COMMENTS**

**2.** Introduction, paragraph 1: This is somewhat inaccurate, in the sense that it is the cells in an organism's tissues that are exposed to mutational processes throughout the organism's life, and each cell or tissue develops its own mutational profile. The existence of a "unique mutational profile" thus may be better described as a property of a tissue, organ or tumour: it is not really accurate to say that each human has "his/her unique mutational profile", as this varies widely across tissues (e.g. cells in the blood, skin, liver, and colon have very different mutational spectra), and the differences among individuals also tend to be tissue-specific.

**Response**: We agree that our phrasing was poorly chosen here. We have altered the text to reflect this comment. The text now says “These processes result in a catalog of somatic mutations in the tissue creating…”.

**3.** Introduction, paragraph 3: in the sentence where the motivation for PM signatures is mentioned ("due to it requiring fewer parameters"), it might be appropriate to add a brief note that the assumption of independence between substitution type and flanking bases also limits the representation of patterns where these features are dependent, although this is seen in relatively few COSMIC signatures (e.g. SBS8, SBS25, SBS35).

**Response**:  We have added a sentence addressing this disadvantage of using the independence assumption.  “However, the independence assumption might prevent signatures with dependent bases from being discovered, thereby resulting in fewer signatures.”

**4.** Implementation, paragraph 1: it would be useful to complement the example in Table 1 and Equation 1 with a figure that shows the original PM signature in Table 1, and the equivalent COSMIC signature that results from applying Equation 1 to each of the 96 substitution types. This would help the reader to understand the conversion between both graphical representations, which is shown in later figures.

**Response**: : As requested, we have added a new Figure 1 that shows the signature in Table 1 and the ‘expanded’ signature in COSMIC format to help illustrate Equation 1. 

**5. **Note that reference 5 has an updated version: Omichessan, Severi & Perduca (2019) ^1^.

**Response**: Reference 5 is now updated.

**6.** Note that reference 6 is missing a colon between the author list and title.

**Response**: : The colon has been added to reference 6.

**7.** The "About iMutSig" web page states that "On the GitHub page, you can: - install the iMutSig R pacakge and run it locally." However, on the GitHub page I found no instructions on how the package can be installed and run locally using R and Shiny. While I understand that the main purpose of the platform is to be an online tool, some users may be interested in having a local copy. For example, the availability of the tool seemed somewhat variable: I was able to access the website (https://zhiyang.shinyapps.io/imutsig/) on 17 June, but not on 18 June. Although this might be a rare issue, it highlights the advantage of providing users with an alternative way of accessing the tool in the long term. For example, some simple steps for installation and running could be added as a README.md file on the GitHub repository.

**Response**: Thank you for the suggestion. A new README.md file was added to the GitHub repository ( https://github.com/USCbiostats/iMutSig) providing instructions on how to install the necessary packages and host the app locally. The website failure on June 18 ^th^ resulted from a scheduled automatic downloading procedure which exceeded the server response time. Subsequently, we found that the COSMIC website posted new versions of signatures (v3.1) under a different website, allowing us to remove the automatic download procedure and avoid such an issue in the future.

**8.** Note that, in the platform interface, some of the signature names read "COSIMIC" instead of "COSMIC".

**Response**: Those typos have been corrected.

**OPTIONAL SUGGESTIONS**

**9.** I found it strange that in the tabs "COSMIC to pmsignature" and "pmsignature to COSMIC", the drop-down menus for choosing the COSMIC and PM signatures to compare are located in the middle of the interface, below the top panels that show the chosen signature. It seems to me that it would be more intuitive to place the selection menus at the top of the page, although I might be wrong.

**Response**: The dropdown menu for selection is now at the top of the webpage.   

**10.** The authors might consider extending the user-supplied signature mode to allow the user to input a set of signatures, and then select the signature to compare using a drop-down menu (as in the other comparison modes), as it is likely that users will be interested in analysing sets of signatures, rather than single signatures. However, I understand this might not be straightforward to implement.

**Response**: Thank you for the suggestion. Indeed, allowing multiple signature inputs will require introducing a drop-down menu in order to maintain the current layout. Although not incorporated at this time, we plan to add this feature in the future. 

**11.** Another potential extension could be an additional mode in which comparison could be performed between two sets of user-supplied signatures (each of which could be in COSMIC or PM format), in order to find the best one-to-one match between the signatures.

**Response**: Thank you for this suggestion as well. In the future, we also plan to add this feature by adding a tab allowing the comparison between user-supplied signatures.
